# Integrating phenotypic analyses and color parameters: a multidimensional framework for precise color characterization in eggplant fruit

**DOI:** 10.3389/fpls.2025.1689896

**Published:** 2025-12-10

**Authors:** Yiying Zhang, Hairong Chen, Shan Deng, Qujiang Bai, Yu Zhang, Hong Zhao, Kun Liu, Yunxia Chu, Li Ren

**Affiliations:** 1 Institute for Agri-Food Standards and Testing Technology, Shanghai Academy of Agricultural Sciences, Shanghai, China; 2 Shanghai Sub-Center for Plant New Variety Tests, Ministry of Agriculture and Rural Affairs, Shanghai, China; 3 College of Agriculture, Anshun University, Anshun, China

**Keywords:** eggplant, CIELAB color parameters, color quantification, DUS test, SSR markers

## Abstract

The accurate quantification of plant organ color remains a major challenge in plant variety identification, particularly when adjacent expression states exhibit subtle visual differences in color. This study addressed this challenge by integrating colorimetric, phenotypic, and genomic analyses of 137 eggplant germplasm resources to characterize their fruit color. The CIELAB color parameters accurately represented fruit coloration and exhibited strong correlations with DNA fingerprinting results, while also aligning with the visual description of color characteristics based on Distinctness, Uniformity, and Stability (DUS) test guidelines. The color transition from harvest maturity to physiological ripeness was effectively captured by shifts in these values. Furthermore, the purple fruits at harvest maturity were subdivided into violet and red subcategories based on their CIELAB parameter distributions, and the yellow, ochre, and brown fruits at physiological ripeness were clearly separated using K-means clustering. Consequently, this study defined precise CIELAB ranges for each color category, offering a robust, multidimensional approach for the objective identification of eggplant varieties and enhancing the reproducibility of color-based DUS evaluations.

## 1 Introduction

The protection of new plant varieties is a key component of intellectual property rights (IPR) (Buanec, 2006). In most countries, distinctness, uniformity, and stability (DUS) serve as the fundamental prerequisites for variety protection and registration (Deng et al., 2019). The DUS test differentiates varieties by evaluating their unique morphological and physiological traits and comparing them with the most similar existing varieties through field or laboratory trials conducted under standardized test guidelines (Wäldchen et al., 2018b; Chu et al., 2023). Consequently, the analysis of DUS characteristics is critical not only for determining varietal distinctness but also for facilitating the breeding of new varieties (Pilarczyk et al., 2015; Wang et al., 2022).

Traditionally, variety identification, which is the foundation of the DUS test, has relied on agronomic and morphological traits. However, the increasing demand for rapid and precise identification methods, driven by both research and commercial needs, has highlighted the limitations of conventional approaches (Tran-Dinh et al., 2009). In this context, DNA fingerprinting has emerged as a powerful tool that significantly enhances the accuracy and efficiency of varietal discrimination (Xie et al., 2019). Over the past decade, China has progressively incorporated DNA fingerprinting into the DUS framework to support variety identification in seed certification. For instance, a recent study demonstrated a strong concordance between DUS results and SNP fingerprinting in cucumbers (Zhang et al., 2022). Despite these advances, color-related traits remain difficult to quantify objectively, particularly when discriminating between adjacent states. The Commission Internationale de l’Éclairage (CIE) LAB color space (Commission Internationale de l’Éclairage, 1978) offers a standardized framework for quantitative color measurements (Darrigues et al., 2008). The CIELAB system has been widely used for color classification in ornamental plants, such as *Tulipa* spp (Torskangerpoll et al., 2005), *Chrysanthemum* (Hong et al., 2012), and *Ranunculus asiaticus* L (Liu et al., 2019), as well as in food quality assessments of plums (Rampáčková et al., 2021), coffee beans (Kulapichitr et al., 2022), and dragon fruit (Permana et al., 2025). Integrating quantitative CIELAB parameters with phenotypic and genotypic data, such as SSR markers, can substantially improve the accuracy and efficiency of variety identification.

Eggplant (*Solanum melongena* L.), a major solanaceous crop, is cultivated worldwide for its nutrient-rich fruits (Gürbüz et al., 2018; Toppino et al., 2020). Fruit skin color, a major determinant of market appeal and economic value, varies widely among cultivars, primarily encompassing white, green, and purple hues. The commercial preference for particular colors differs across regions, particularly in China, where consumer demand strongly shapes the market value (Zhou et al., 2022). The color of eggplant fruit is largely governed by the type and concentration of anthocyanins and chlorophyll (Zhou et al., 2021). However, continuous variations in pigmentation often blur the boundaries between adjacent color categories, complicating visual identification. Thus, developing a quantitative and objective method to characterize the color of eggplant fruits is essential for breeding programs and DUS testing.

This study aimed to precisely define eggplant fruit skin color phenotypes and elucidate the relationship between subjective visual evaluation and instrumental color measurements. Using a colorimeter, we recorded the CIELAB color parameters from the fruit surface and analyzed the data in conjunction with the SSR marker profiles. Our objective was to establish a quantitative standard for fruit color characterization to enhance the objectivity and reproducibility of the DUS test.

## 2 Materials and methods

### 2.1 Eggplant varieties and DUS characteristics

A total of 137 eggplant germplasm resources were obtained from the Shanghai Sub-Center for Plant New Variety Tests of the Ministry of Agriculture and Rural Affairs, China. The plants were cultivated in a greenhouse in Shanghai. Each accession was sown on 23 February 2023, and a minimum of 30 plants per accession were maintained for sampling.

**Figure 1:**
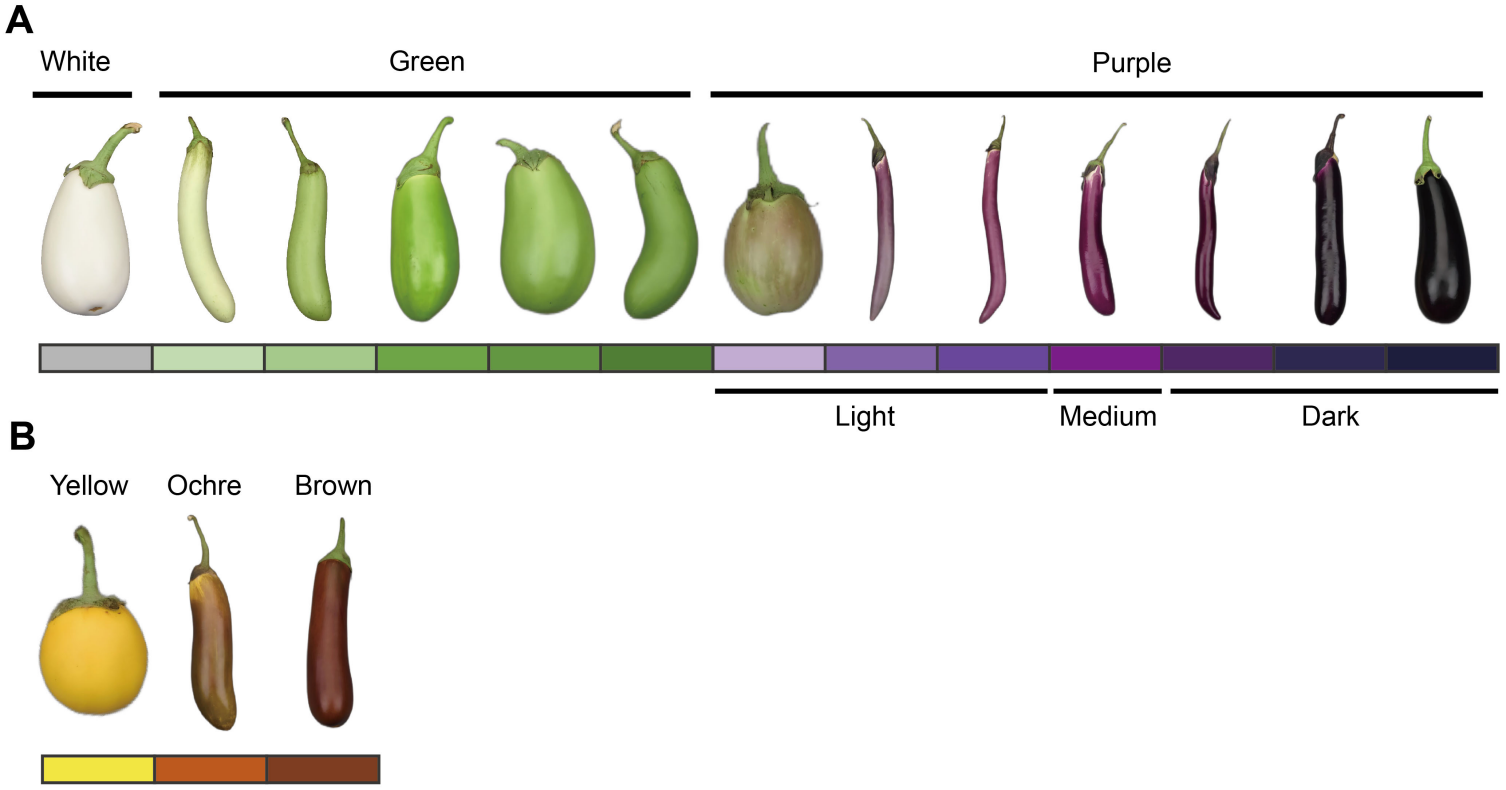
Schematic diagram of the eggplant fruit skin main color characterization at different stages. **(A)** Main color of the fruit skin at harvest maturity (FSCH) and intensity of the main color of the fruit skin (IFC). **(B)** Fruit skin color at physiological ripeness (FSCP). The colored blocks correspond to the type and intensity of the fruit skin color. These colored blocks were set up throughout the full text of the corresponding images. According to the value assignment criteria in the eggplant DUS test (**Supplementary Table S1**), the color intensity of purple fruit skin was classified as light (notes 1–4), medium (notes 5 and 6), and dark (notes 7–9). The eggplant fruits in the figure are only an indication of color and do not represent the proper proportion of fruit size.

Ten DUS test characteristics related to organ color and anthocyanin pigmentation were assessed according to the national and international guidelines for eggplants (Li et al., 2017; International Union for the Protection of New Varieties of Plants [UPOV], 2002). These characteristics included anthocyanin coloration of hypocotyl (ACH), anthocyanin coloration of stem (ACS), intensity of leaf blade green color (ILG), anthocyanin coloration of leaf vein (ACL), flower color (FC), fruit pulp color (FPC, reflecting chlorophyll accumulation in the underlying pulp), fruit skin main color at harvest maturity (FSCH), intensity of fruit skin main color (IFC, evaluated only for green and violet varieties), fruit glossiness (FG), and fruit skin color at physiological ripeness (FSCP). All traits were evaluated through group visual observation, and the scoring criteria are presented in **Supplementary Table S1**. The three principal fruit color characteristics—FSCH, IFC, and FSCP—are illustrated in **Figure 1**.

### 2.2 Acquisition and calculation of color parameters

Fruit skin color was quantified using a handheld colorimeter (CR-400, Konica Minolta, Japan) to obtain the CIELAB color values. For each accession, fruits from five randomly selected plants were measured at both harvest maturity and at physiological ripeness. The colorimeter was calibrated using a standard white plate before measurement. A single reading was taken for each fruit at a uniform, defect-free area of the skin. The colorimeter directly provided the values of L^*^ (lightness, from 0 [black] to 100 [white]), a^*^ (from green [−*a*] to red [+*a*]), and b^*^ (from blue [−*b*] to yellow [+*b*]) values. Chroma (C^*^), hue (H^*^), and total color difference (ΔE^*^) were calculated using standard formulas.


$$C^{*}={\left(a^{*2}+b^{*2}\right)}^{1/2}$$

$$H^{*}=tan^{-1}\left(b^{*}/a^{*}\right),$$

$$\Delta E^{*}={\left(\Delta L^{*2}+\Delta a^{*2}+\Delta b^{*2}\right)}^{1/2}.$$

where ΔL^*^, Δa^*^, and Δb^*^were calculated as the difference in L^*^, a^*^, and b^*^ values, respectively, between harvest maturity and physiological ripeness stages.

### 2.3 DNA extraction and SSR genotyping

Genomic DNA was extracted from fresh leaf tissue using the CTAB method (Murray and Thompson, 1980). Twenty-four polymorphic SSR primer pairs were selected to ensure even coverage across all 12 eggplant linkage groups (**Supplementary Table S2**), providing a genome-wide representation for association analysis. Fluorescently labeled DNA fragments were automatically sized, and the resulting data were statistically analyzed.

### 2.4 Analysis of association

The population structure of the 137 germplasm resources was assessed using STRUCTURE 2.3.1 (Pritchard et al., 2000), which applies a Bayesian clustering algorithm. The number of assumed populations (K) ranged from 1 to 15, with five independent runs per K. Each run consisted of a burn-in period of 10,000 steps, followed by 100,000 Markov Chain Monte Carlo (MCMC) iterations. The optimal K value was determined by evaluating both the estimated log probability of the data [LnP(D)] (Pritchard et al., 2000) and Delta K (ΔK) (Evanno et al., 2005). Marker–trait associations between SSR markers and 16 traits (ten DUS characteristics and three color parameters measured at two developmental stages) were analyzed using the general linear model (GLM) implemented in Tassel 5. The Q-matrix representing the population structure was incorporated as a covariate to correct for stratification. Associations were considered highly significant at *P*<0.001, and for each significant marker, the proportion of phenotypic variance explained was estimated (Bradbury et al., 2007).

### 2.5 Statistical analysis and visualization

Statistical analyses and data visualizations were performed using Origin 2024 (OriginLab Corporation, USA). Pearson’s correlation coefficients were calculated to the evaluate relationships among the 10 DUS test characteristics and three color parameters measured at two developmental stages. Three-dimensional (3D) scatter plots and two-dimensional (2D) projection plots of the L^*^, a^*^, and b^*^ values were generated to visualize the distribution of the CIELAB color parameters across both stages. Lollipop charts were used to depict temporal changes in fruit color from harvest maturity to physiological ripeness, based on shifts in the L^*^, a^*^, and b^*^ values. K-means clustering was applied to the color parameters, with the optimal cluster number (K) determined using the Elbow Method and the Silhouette Coefficient. Principal component analysis (PCA) was conducted to visualize the clustering structure. The distribution of purple fruits is represented in a score plot based on the first two principal components (PC1 and PC2). To define the color range of each eggplant color group, gradient color blocks were rendered by converting the L^*^, a^*^, and b^*^ values into RGB format (https://www.colortell.com/labto) and composed in Adobe Photoshop CC (2019) to illustrate the representative hues of each major cluster.

## 3 Results

### 3.1 Correlation among color parameters of fruit, DUS test characteristics, and SSR markers

**Figure 2:**
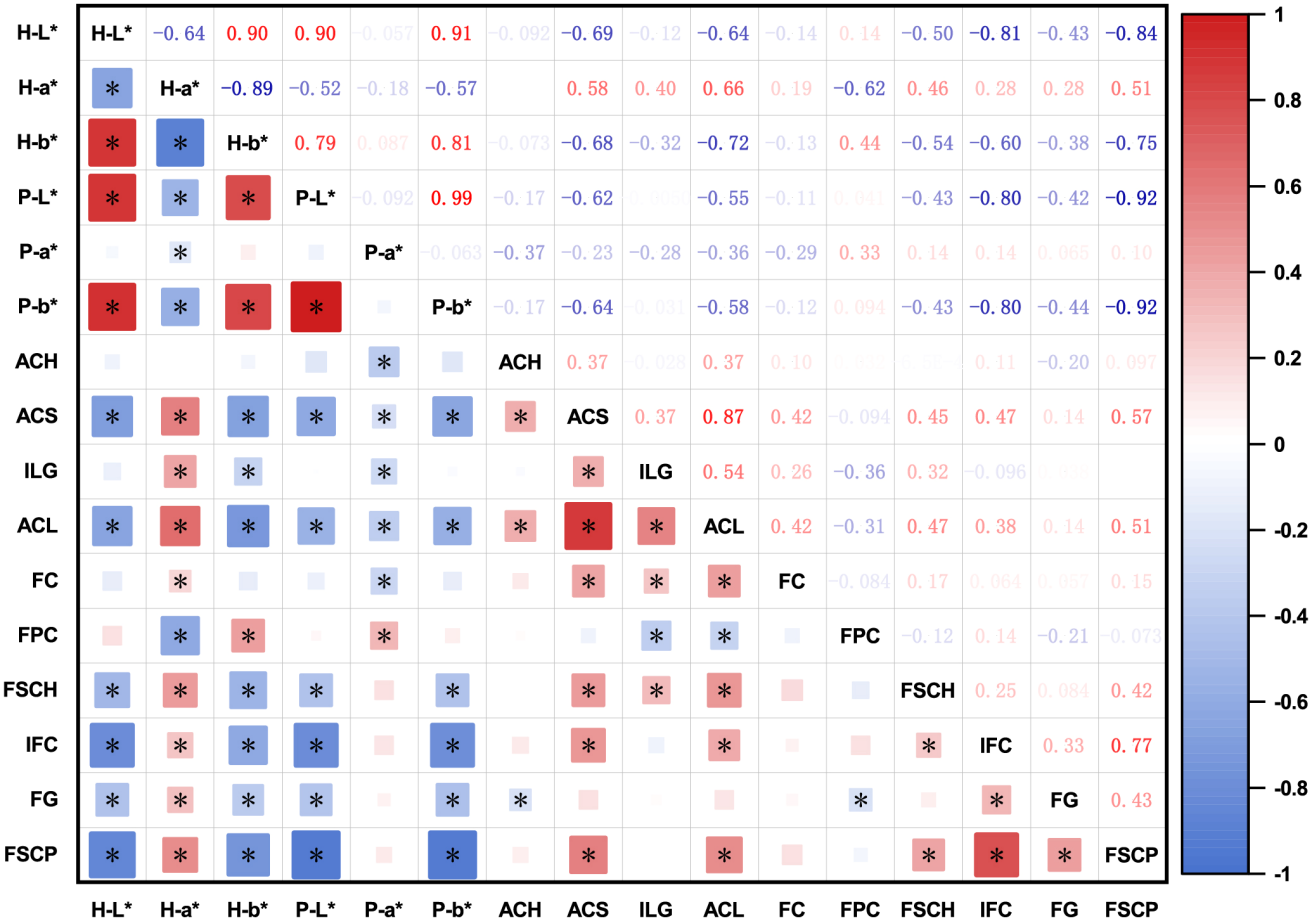
Correlation matrix between color parameters and DUS test characteristics of eggplant. The areas and colors of the rectangles represent the absolute values of the corresponding correlation coefficients. Darker red denotes a higher positive correlation and darker blue denotes a higher negative correlation. Correlation coefficients significant at the 0.05 level are denoted by *. The color parameters of eggplant fruit at harvest maturity or physiological ripeness were expressed as H-L*, H-a*, H-b*, P-L*, P-a*, and P-b*. ACH, anthocyanin coloration of hypocotyl; ACS, anthocyanin coloration of the stem; ILG, intensity of leaf blade green color; ACL, anthocyanin coloration of the leaf vein; FC, flower color; FPC, fruit pulp color; FSCH, fruit skin main color at harvest maturity; IFC, intensity of fruit skin main color; FG, fruit glossiness; FSCP, fruit skin color at physiological ripeness.

The bivariate correlations between the color parameters (L^*^, a^*^, and b^*^) and DUS characteristics are shown in **Figure 2**. Overall, positive correlations were observed between both color parameters and DUS traits. Among the color parameters, the a* value at harvest maturity (H-a*) exhibited negative correlations with other color parameters. Among the DUS characteristics, ACS and ACL were strongly correlated with most of the other characteristics. The high positive correlation between ACS and ACL implies synchronized anthocyanin metabolism across multiple eggplant organs. Additionally, IFC was positively correlated with FSCP, suggesting that a darker fruit color at harvest maturity corresponds to a darker coloration at physiological ripeness. Significant negative correlations were observed between the color parameters and DUS characteristics. In particular, ACS, ACL, IFC, and FSCP were significantly correlated with all color parameters, confirming that anthocyanin accumulation in eggplant organs directly influences color. FPC was significantly negatively correlated with H-a*, possibly reflecting the influence of pulp color on the visual appearance of the fruit skin color.

Bayesian clustering analysis identified a peak ΔK value at K = 2 (**Supplementary Figure S1**), indicating that the 137 germplasm accessions could be classified into two principal subpopulations. In total, 24 highly significant marker–trait associations (*p <*0.001) were detected for the three color parameters and ten DUS characteristics (**Table 1**). The phenotypic variance explained (R²) by the individual markers ranged from 6% to 44%. Five markers—emx30903, emx40203, emx50304, emx81003, and emxC0805—exhibited strong associations with color parameters and anthocyanin pigmentation, indicating their key role in color expression. Conversely, fewer significant associations were identified for P-a*, ACH, FC, and FG, which is consistent with their weaker phenotypic correlations (**Figure 2**). Together, these results provide genetic evidence for a strong association between anthocyanin biosynthesis and color traits, suggesting possible pleiotropy or genetic linkage among the underlying loci.

**Table 1:** SSR markers associated with traits (*p* < 0.001) and their explained phenotypic variation.

SSR markers	Color parameters						DUS test characteristics									
	H-L*	H-a*	H-b*	P-L*	P-a*	P-b*	ACH	ACS	ILG	ACL	FC	FPC	FSCH	IFC	FG	FSCP
emb01J19	0.08	0.11	0.09	0.11		0.10						0.18	0.09	0.16	0.09	0.10
eme07D02					0.07				0.10						0.09	
emf01E17									0.11			0.09				
emg11B20									0.18					0.10		
emg11D05	0.10		0.11	0.11		0.13		0.08		0.07			0.11	0.10	0.10	0.09
emh01O20		0.11							0.12			0.19				
emh02A04										0.07						
emh02E08																
emh11j22			0.12			0.09		0.09		0.12		0.15	0.09	0.10		
emx30204	0.12	0.16	0.10						0.13			0.13	0.09	0.11	0.09	
emx30903		0.18	**0.31**	0.13		0.15	0.06	0.10	0.09	0.14		0.15				0.13
emx40203	0.19	**0.32**	**0.44**	**0.21**		**0.23**		**0.25**	0.14	**0.30**			**0.20**	0.12		0.19
emx50304	**0.30**	**0.23**	**0.30**	0.17		0.17		0.14	0.13	**0.22**		0.18	**0.31**	**0.27**		0.15
emx50804				0.13		0.13			0.12					0.11	0.12	0.15
emx60604									0.17							
emx61102				0.09		0.09			**0.27**					0.12		0.10
emx70203	0.15						0.06		0.10				0.15	0.13		
emx81003		**0.21**	**0.25**	0.10	0.06	0.10	0.06		0.19	0.12		0.11		0.13		0.10
emx91003					0.06		0.12									
emxA0405									0.18	0.08					0.09	
emxA0703	0.09	0.12	0.14	0.12	0.06	0.13	0.06		0.10	0.08		0.11	0.08	0.11	0.11	0.14
emxB1002		0.08	0.10					0.08		0.11		0.17		0.10	0.08	
emxC0805	0.10	**0.27**	**0.35**	0.16		0.18		0.13	0.18	**0.20**	0.07		0.11	0.16		0.16
emxC1103	0.11		0.10	0.16		0.16		0.08								0.14

The bold values indicated the explained phenotypic variation greater than 20%.

The color parameters of eggplant fruit at harvest maturity or physiological ripeness were expressed as H-L*, H-a*, H-b*, P-L*, P-a*, P-b*, respectively. ACH: anthocyanin coloration of hypocotyl; ACS, anthocyanin coloration of the stem; ILG, intensity of leaf blade green color; ACL, anthocyanin coloration of the leaf vein; FC, flower color; FPC, fruit pulp color; FSCH, fruit skin main color at harvest maturity; IFC, intensity of fruit skin main color; FG, fruit glossiness; FSCP, fruit skin color at physiological ripeness. The underlined numbers indicated explained a phenotypic variation greater than 15%, and the underlined and bolded numbers indicated explained phenotypic variation greater than 20%.

### 3.2 Distribution of CIELAB color parameters of eggplant fruit color

**Figure 3:**
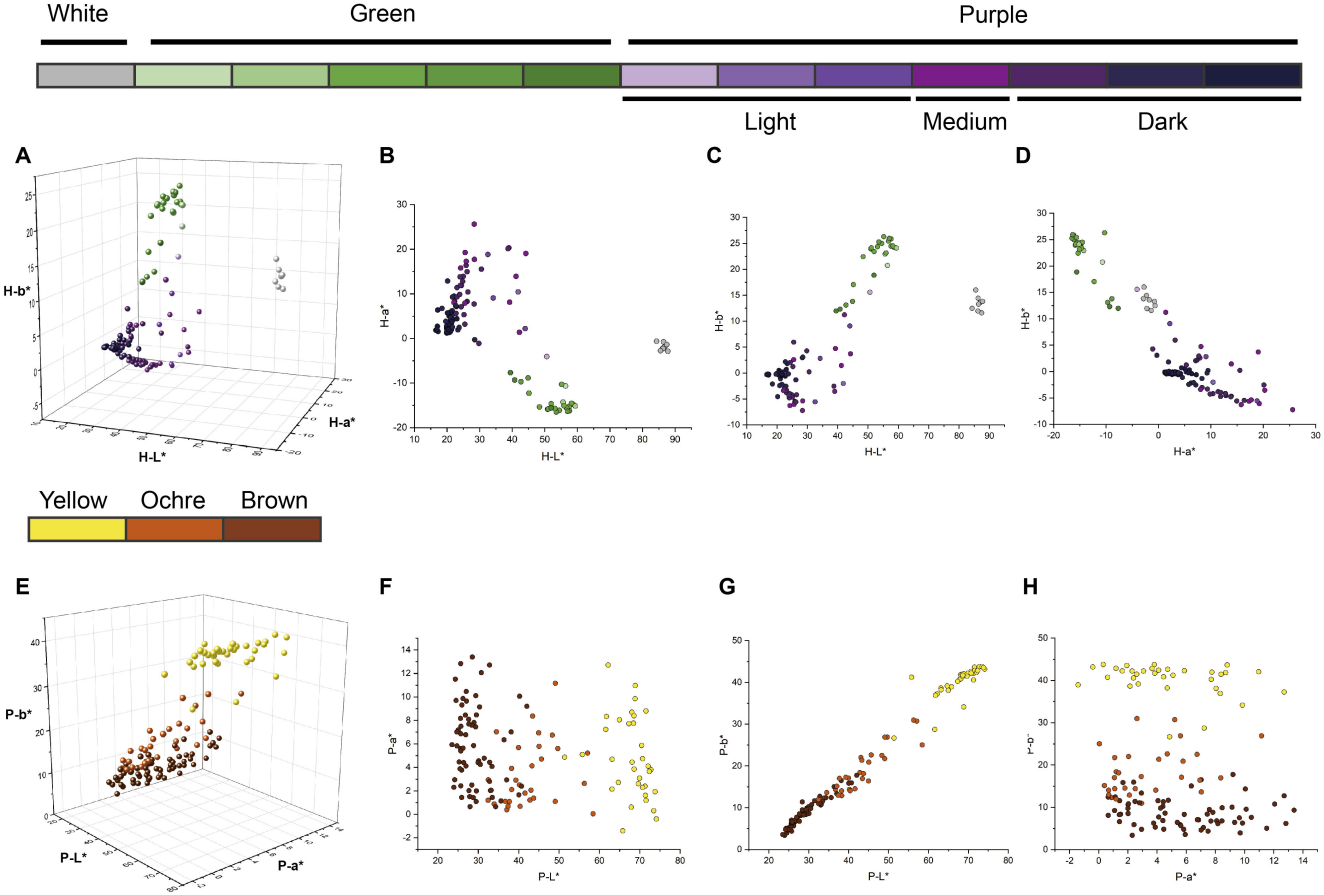
Distribution diagrams of L^*^, a^*^, and b^*^ values of different colors and color intensities of harvest-maturity fruits and physiological ripeness fruits. The colors of the dots correspond to the types of eggplant fruit colors indicated by the same color block as in **Figure 1**. **(A)** 3D distribution diagrams of the L^*^, a^*^, and b^*^ values of harvest-maturity fruits. **(B–D)** Distribution diagrams of the L^*^, a^*^, and b^*^ values of harvest-maturity fruits in the 2D projection. **(E)** 3D distribution diagrams of the L^*^, a^*^, and b^*^ values of the physiological ripeness fruits. **(F–H)** Distribution diagrams of the L^*^, a^*^, and b^*^ values of physiological ripeness fruits in the 2D projection.

The L^*^, a^*^, and b^*^ values of the eggplant fruit skin varied with pulp color and developmental stage (harvest maturity versus physiological ripeness). At harvest maturity, distinct boundaries between color groups were evident in the 2D CIELAB plots (**Figure 3**). Thresholds of L^*^ = 70 and a^*^ = *−*5 effectively separated white-, purple-, and green-fruited accessions (**Figure 3B**). However, three light-purple accessions overlapped with green-fruited accessions because of their green pulp, which influenced the overall color parameters (**Figure 3C**). At physiological ripeness, the CIELAB distributions clearly distinguished between the yellow and ochre fruits, whereas the boundary between the ochre and brown groups was less distinct (**Figures 3E–H**). Fruits near these boundaries often displayed striped or nonuniform pigmentation. These results demonstrate that the CIELAB color space provides an effective and objective framework for classifying the color of eggplant fruits across developmental stages.

### 3.3 Temporal change traits of eggplant fruit coloration

**Figure 4:**
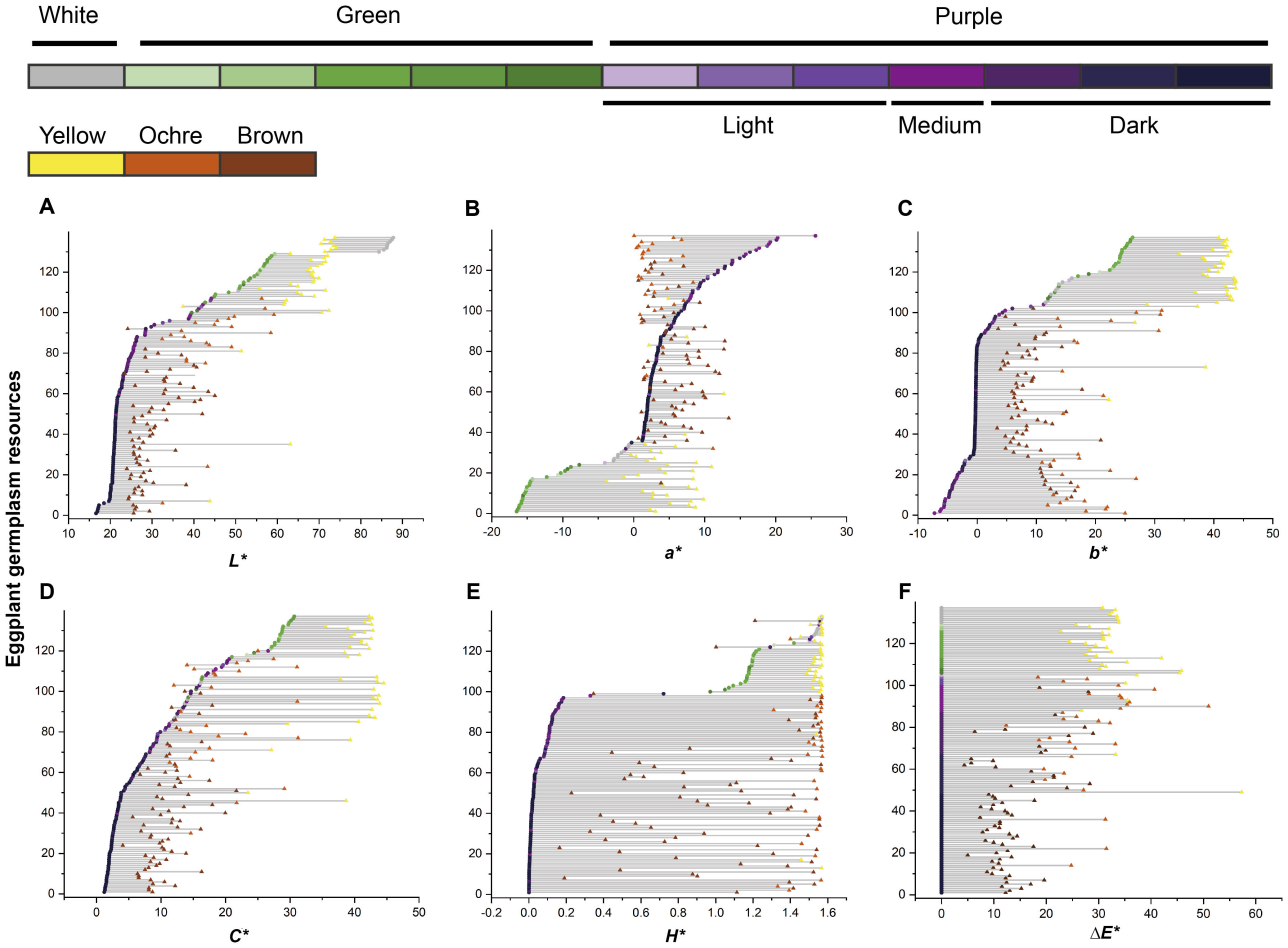
Temporal changes in eggplant fruit color parameters. The vertical axis represents the serial number of the eggplant resource samples. **(A–E)** The arrangement orders of the samples were sorted in increasing order according to the values of L^*^, a^*^, b^*^, C^*^, and H^*^ at harvest maturity. **(F)** The arrangement orders of the samples were sorted in descending order according to the color intensity of the harvest-maturity fruits. Dots represent harvest-maturity fruits, and triangles represent physiological ripeness fruits. The colors of the dots and triangles correspond to the types of eggplant fruit colors indicated by the same color blocks as in **Figure 1**.

Changes in the color parameters of the eggplant fruit were analyzed from harvest maturity to physiological ripeness (**Figure 4**). Fruits with white or green skin at harvest maturity turn yellow upon ripening, whereas purple-skinned fruits generally develop ochre or brown hues. A positive correlation was observed between the intensity of purple coloration at harvest maturity and the degree of browning at physiological ripeness, indicating that the anthocyanin content at harvest maturity influenced the final ripened color. Specifically, the L^*^ values of purple and green fruits increased, whereas those of white fruits decreased from harvest maturity to physiological ripeness (**Figure 4A**). The changes in a^*^, b^*^, C^*^, and ΔE^*^ values across the two stages were more pronounced in light and medium purple, green, and white fruits than in dark purple fruits (**Figures 4B–D, F**). Conversely, variations in the H^*^ values were greater in light- and medium-purple fruits than in dark purple, green, and white fruits (**Figure 4E**).

### 3.4 Refining the classification of eggplant fruit color based on CIELAB color parameters

**Figure 5:**
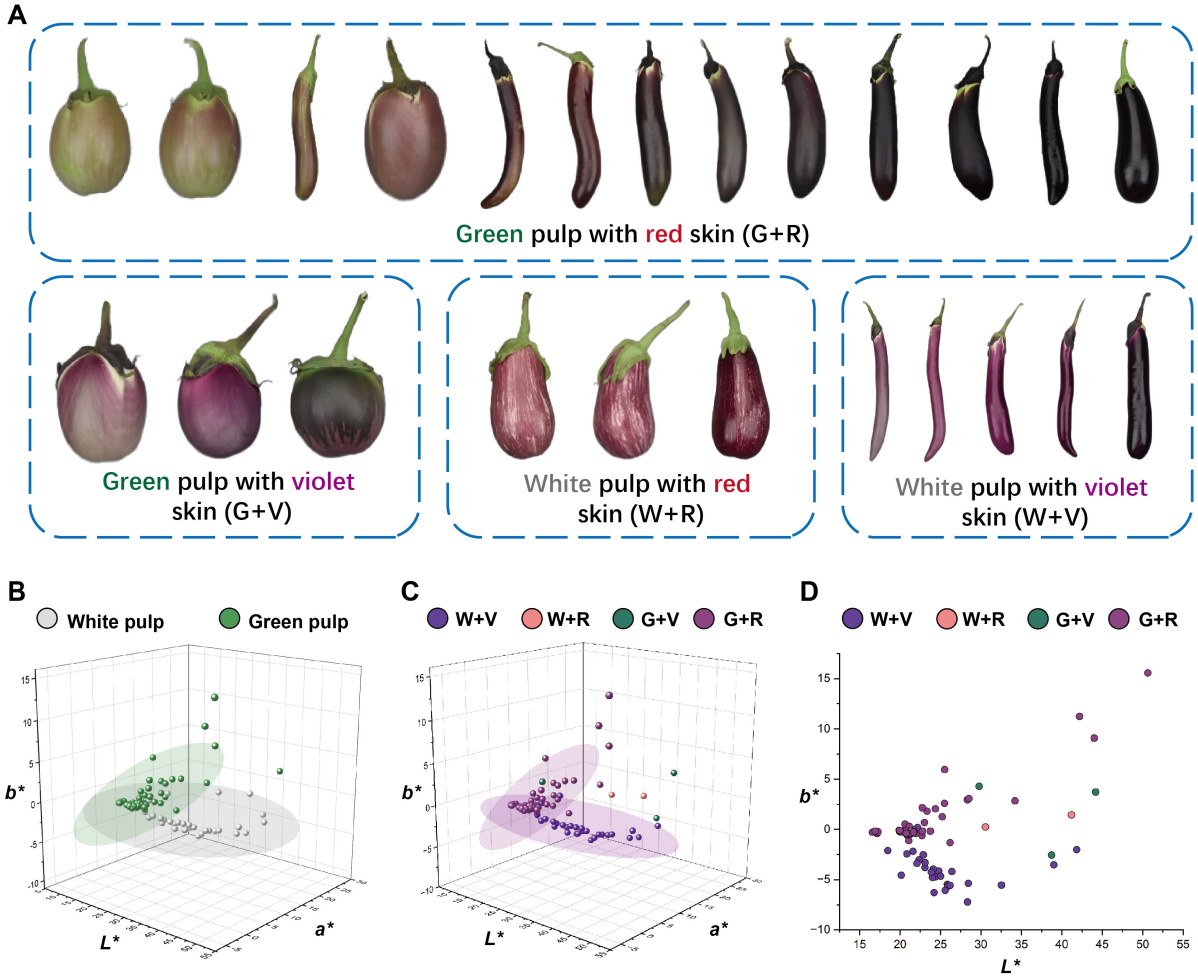
Artificially divided four categories of purple fruit morphology and the distribution diagrams of the L^*^, a^*^, and b^*^ values. **(A)** Schematic representation of the four categories of purple fruits. The eggplant fruits in the figure are only an indication of color and do not represent the true proportion of fruit size. **(B)** Distribution diagrams of the L^*^, a^*^, and b^*^ values of the white and green pulp of purple fruits. **(C)** Distribution diagrams of the L^*^, a^*^, and b^*^ values of the four categories of purple fruits. **(D)** Distribution diagrams of the four categories of purple fruits in the 2D coordinates of L^*^ and b^*^ values.

As noted above, the visual appearance of purple fruits is determined by both anthocyanin accumulation in the skin and chlorophyll retention in the underlying pulp, resulting in a wide spectrum of purple phenotypes. Representative examples of these categories are shown in **Figure 5A**. Based on visual observations, the purple fruits were artificially classified into four groups: green pulp with red skin (G + R), green pulp with violet skin (G + V), white pulp with red skin (W + R), and white pulp with violet skin (W + V). To further quantify the relative contributions of pulp and skin color, the L^*^, a^*^, and b^*^ values were plotted separately in CIELAB color space. Two distinct confidence ellipses emerged in the 3D plots of pulp color (**Figure 5B**) and skin color (**Figure 5C**). As shown in **Figure 5D**, the categories of purple fruits were largely distinguishable along the b-axis, with red and violet fruits separated by an approximate boundary at b^*^ = −2.5. These findings demonstrate that the purple hue of eggplant fruits depends on both pulp color and the specific types of anthocyanins, suggesting that the DUS classification for purple fruit color should be further subdivided.

**Figure 6:**
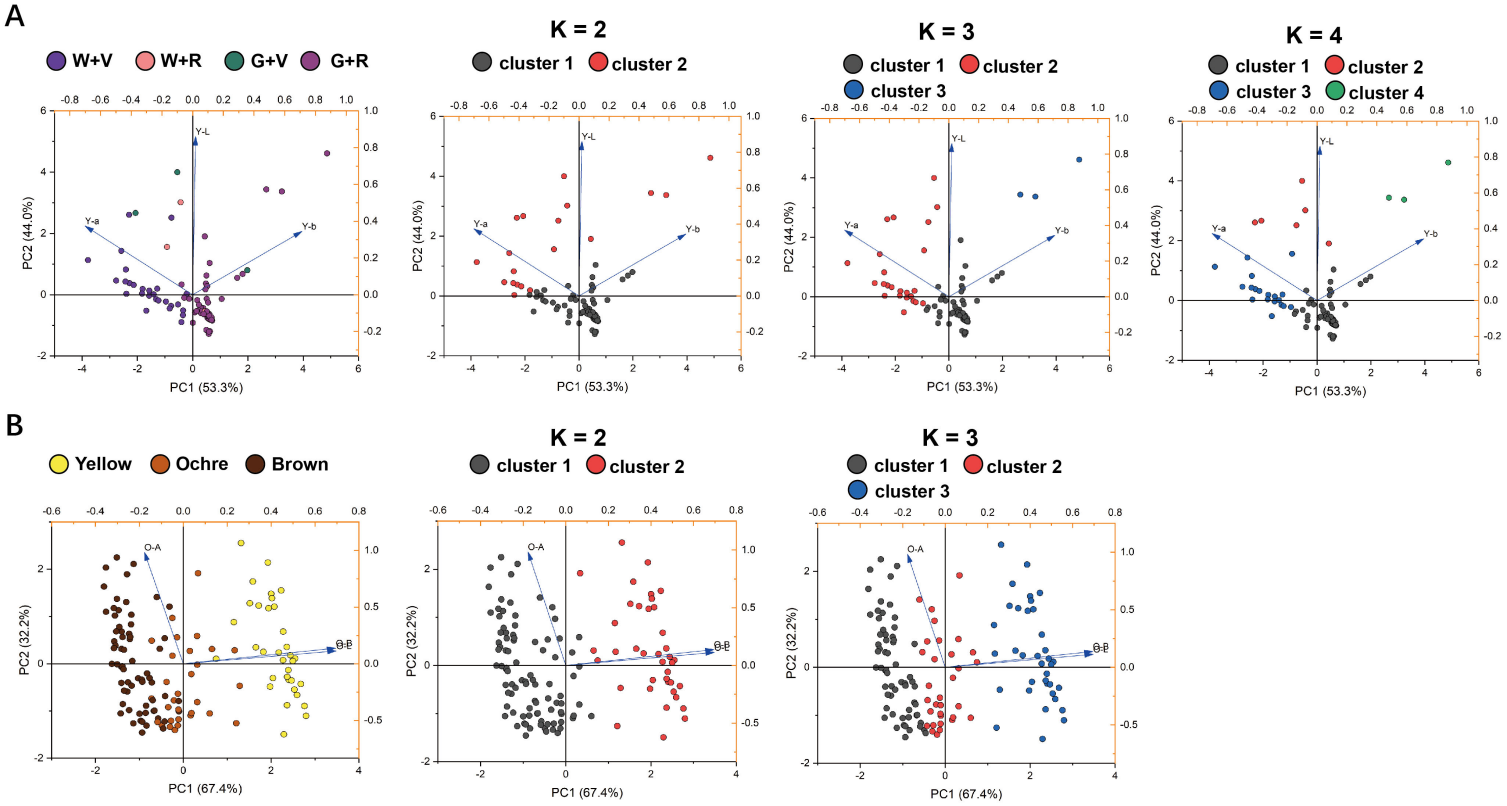
Distribution of eggplant fruits on score plots according to the first and second principal component scores. The classification elements were the L*, a*, and b* values. **(A)** The leftmost column of images shows the result of artificial classification of the four categories of purple fruits at harvest maturity, and the three columns of images on the right show the result of K-means classification. **(B)** The leftmost column of images shows the result of artificial classification of the fruits at physiological ripeness, and the two columns of images on the right show the result of K-means classification.

The correspondence between the artificial and K-means classifications was visualized using principal component score plots (**Figure 6**). This comparison aimed to determine whether the unsupervised K-means algorithm could replicate expert-based morphological classifications or identify alternative data-driven groupings from the CIELAB parameters. The optimal cluster number (K) determined by the Elbow Method and Silhouette Coefficient was two or three for the harvest maturity stage of purple fruits and the physiological ripeness stage of all germplasm accessions (**Supplementary Figure S2**). Given that the artificial classification divided purple fruits into four categories (**Figure 5**), K values of 2, 3, and 4 were tested at the harvest-maturity stage.

At harvest maturity (**Figure 6A**), K = 2 separated purple fruits into dark and light/medium purple categories based on color intensity. When K = 3, cluster 1 corresponded closely to the artificial G + R category, whereas fruits categorized as W + V, W + R, and G + V were merged into cluster 2. For K = 4, clusters 1 and 2 matched the G + R and W + V groups, respectively, but clusters 3 and 4 did not align with W + R and G + V groups. Overall, K-means clustering was primarily driven by the type and intensity of skin color, whereas artificial classification accounted for both skin and pulp colors. Although the two methods yielded partially inconsistent results, both revealed two distinct color types among purple fruits at harvest maturity. Consequently, the “purple” state of the eggplant fruit skin color can be rationally subdivided into violet and red.

At the physiological ripeness stage (**Figure 6B**), K = 2 effectively isolated the yellow fruits (cluster 2) but grouped the ochre and brown fruits (cluster 1). This issue was addressed when setting K = 3, which produced a clear separation into three distinct clusters that closely matched the visual classification: brown (cluster 1), ochre (cluster 2), and yellow (cluster 3). Therefore, the three-cluster (K = 3) configuration provided the most accurate correspondence with the visual assessment based on the characteristic states defined in the DUS test.

### 3.5 Various types of eggplant fruit color range and visualization

**Figure 7:**
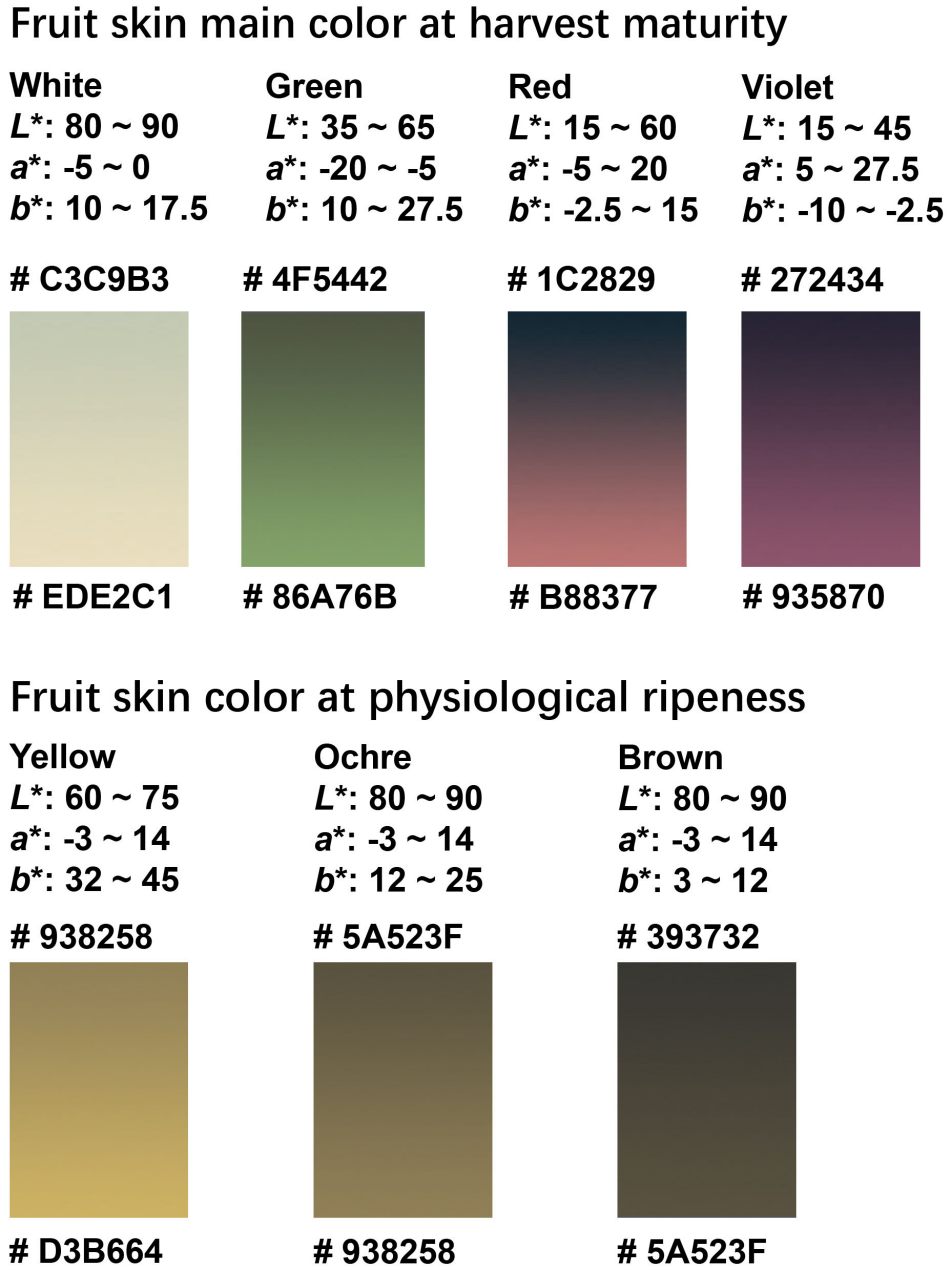
Gradient color blocks used to describe the color range of fruit-color traits at harvest maturity and physiological ripeness. The gradients were formed based on the range of L^*^, a^*^, and b^*^ values of each color type, and their respective RGB codes were marked.

Statistical analyses revealed a continuous variation in the eggplant fruit color, as quantified by the CIELAB color parameters. Accordingly, we summarized the ranges of the L*, a*, and b* values defining each color category. At harvest maturity, the white, green, and purple fruits were clearly distinguished in the CIELAB color space. Moreover, the CIELAB model effectively differentiated between fruits containing red and violet anthocyanins. At physiological ripeness, distinguishing between the yellow, ochre, and brown categories was more challenging owing to the overlapping distributions of their L^*^, a^*^, and b^*^ values. Nevertheless, K-means clustering successfully discriminated between these three color groups and minimized the subjective biases inherent in visual evaluation. By integrating the results from the CIELAB parameter distributions and K-means classifications, we constructed gradient color blocks corresponding to each category based on the L^*^, a^*^, and b^*^ coordinates (**Figure 7**). Collectively, these findings establish a quantitative framework for fruit color characterization and provide new insights for the refinement of purple category subdivisions in DUS tests, beyond visual assessment.

## 4 Discussion

### 4.1 Differences in anthocyanins of purple eggplant fruits

Anthocyanins, the principal water-soluble pigments in plants, are key determinants of plant coloration (Gould, 2004; Chen et al., 2019). The biosynthetic and regulatory mechanisms of anthocyanins differ widely across species (Wu et al., 2016; Guo et al., 2019; Colanero et al., 2020), resulting in red, blue, and purple hues in plant tissues (Lightbourn et al., 2008; Lu et al., 2015; Li et al., 2020). In this study, red and violet eggplant fruits were clearly differentiated using CIELAB color parameters (**Figures 5C, D**), suggesting that variations in purple pigmentation are linked to distinct anthocyanin synthesis pathways.

This study established a quantitative CIELAB-based approach that objectively distinguished red and violet eggplant fruits within the DUS framework. Prior research supports the need for this refinement. Zhou et al. (2022) demonstrated that white and green eggplants contain cyanidin derivatives but lack delphinidin derivatives, and that pelargonidin-type anthocyanins contribute to red-to-black-purple pigmentation in eggplant skin. Similarly, Yang et al. (2022) expanded the traditional purple color category to include violet and red, showing that red eggplants contain higher delphinidin levels than violet eggplants. Building on these findings, our results highlight the importance of further exploring the pigment composition, genetic regulation, and dynamic changes during fruit ripening that underlie the color variation between violet and red eggplants.

Higher anthocyanin concentrations generally produce a deeper purple hue in eggplant skin (Yang et al., 2022). However, at low concentrations, anthocyanins cannot fully mask the green coloration caused by chlorophylls. Thus, the overall plant coloration depends on the relative proportions of various pigments (Guo et al., 2019; Feng et al., 2021). In this study, the three light-purple eggplant germplasms were positioned near the green-fruit group in the CIELAB color space (**Figure 3C**). Each exhibited a green pulp, suggesting that the pulp color significantly influenced the color parameters of the light-purple fruits. Consequently, an accurate and objective description of eggplant skin color requires consideration of both skin and pulp coloration.

### 4.2 New interpretation of eggplant fruit color in the DUS test

The UPOV and China DUS test guidelines categorize eggplant fruit skin color at harvest maturity into three states: white, green, and purple. However, several studies have proposed broader classifications encompassing black-purple, reddish-purple, lavender, white, orange, and green (Sadilova et al., 2006; Todaro et al., 2009; Ferarsa et al., 2018; Yong et al., 2019; Condurache et al., 2021). Using CIELAB quantification, this study revealed a much broader color spectrum than the DUS classifications particularly within the purple category. To enhance the resolution, we defined objective CIELAB thresholds that subdivided purple into violet and red. Similarly, K-means clustering significantly improved the discrimination among visually similar colors, such as ochre and brown, in physiologically ripe fruits.

However, for striped fruits, color determination using CIELAB parameters was strongly influenced by the pulp color. Purple-striped fruits on green backgrounds were mapped between the green and purple clusters, whereas brown-striped fruits on yellow backgrounds were mapped between the yellow and ochre clusters (**Figure 3**). Therefore, the striped eggplant color should be described in terms of both stripe color and pulp color intensity for a comprehensive evaluation.

In summary, we established a quantitative framework for defining eggplant fruit skin color using CIELAB color parameters. Distinct color thresholds were derived from the distribution of data in the CIELAB space and validated using K-means clustering (**Figure 7**). This objective, data-driven approach resolves longstanding ambiguities in color differentiation and provides a more precise and reproducible standard for describing fruit skin color during DUS testing.

### 4.3 Integrating multidimensional phenotypic data for plant variety identification

Association analysis based on linkage disequilibrium identifies correlations between phenotypic traits and genetic markers within natural populations (Saeidnia et al., 2021). Among the 24 SSR markers analyzed, five markers showed significant associations with both fruit color parameters and anthocyanin-related DUS traits. This finding underscores the importance of integrating agronomic traits with molecular fingerprinting for reliable varietal identification. Notably, two SSR markers (emx40203 and emx50304) displayed the strongest correlations with both color parameters and DUS-defined skin color, highlighting their potential as functional markers for identifying skin color genotypes.

This study also demonstrated that quantitative color parameters can serve as novel phenotypic descriptors in DUS testing. We defined ranges for L^*^, a^*^, and b^*^ values corresponding to different skin color types at harvest maturity and physiological ripeness, enabling the subdivision of the purple category into distinct subtypes with different skin colors. Compared with subjective visual assessment, these quantitative parameters provide higher precision and eliminate the ambiguity associated with adjacent color classes.

By digitizing color traits, this computational approach advances plant phenomics by enabling standardized and reproducible color characterization. The increasing integration of computer vision and machine learning in plant phenotyping (Wäldchen and Mäder, 2018a; Malik et al., 2022) makes these quantitative descriptors highly valuable. The CIELAB parameters established in this study provide robust, high-quality input for automated phenotyping systems. Future work could integrate computer vision for real-time color extraction with machine learning algorithms to develop predictive models that classify color and estimate biochemical attributes, such as anthocyanin content, directly from organ-level images, introducing a new quantitative dimension to DUS testing.

## 5 Conclusion

In summary, this study integrated colorimetric, phenotypic, and genomic analyses to investigate fruit color variation in a diverse panel of 137 eggplant germplasm resources. The results revealed strong correlations among the genetic profiles (SSR markers), DUS characteristics, and quantitative color parameters. Specifically, the CIELAB parameters (L^*^, a^*^, and b^*^) accurately represented fruit color and intensity, enabling the delineation of precise color-space intervals for each skin color category at both harvest maturity and physiological ripeness. Furthermore, the transition in fruit color between the two stages was quantitatively characterized. These findings establish a novel, objective framework for describing eggplant fruit color, with direct applications in DUS testing and varietal identification. Notably, the “purple” expression state in the DUS test was further resolved into two subtypes, violet and red, based on CIELAB color analysis, reflecting distinct patterns of anthocyanin synthesis. Future research should elucidate the mechanisms underlying eggplant skin pigmentation, particularly the interactions between anthocyanins and chlorophylls, to refine the scientific basis for expanding DUS test descriptors for eggplants.

## Data Availability

The original contributions presented in the study are included in the article/**Supplementary Material**. Further inquiries can be directed to the corresponding authors.
