# Supplementary material for: Integrating phenotypic analyses and color parameters: a multidimensional framework for precise color characterization in eggplant fruit
Source: Front Plant Sci. 2025 Dec 10;16:1689896. doi: 10.3389/fpls.2025.1689896 (PMC12728077; doi:10.3389/fpls.2025.1689896)
Supplement: Supplementary file 1 [file DataSheet1.docx]

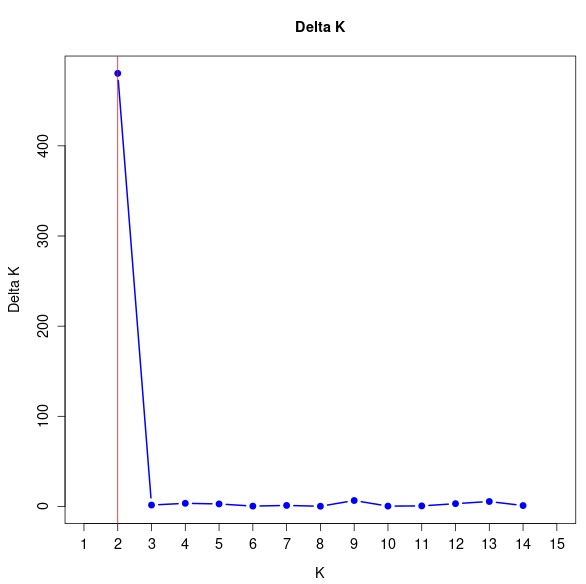

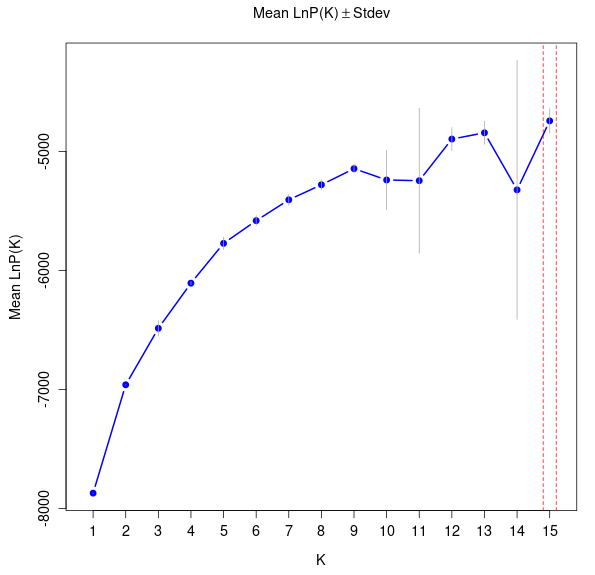


**Figure S1** Variance of an average LnP(D) and ∆K (Delta K) with different values of K.

**Fruit skin main color at harvest maturity**


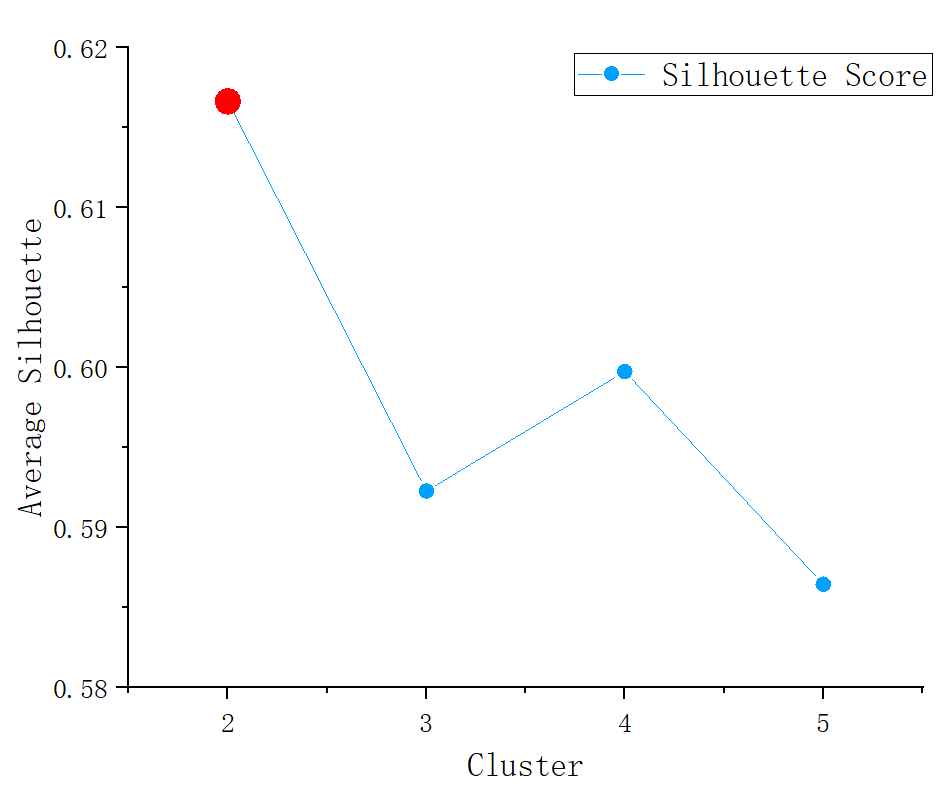


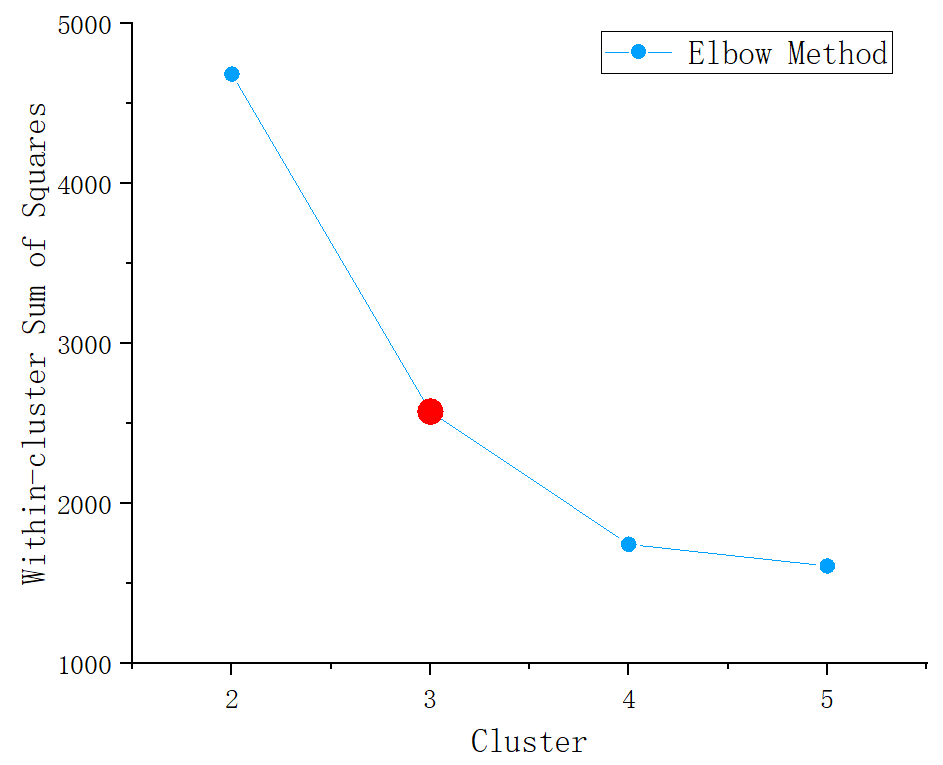


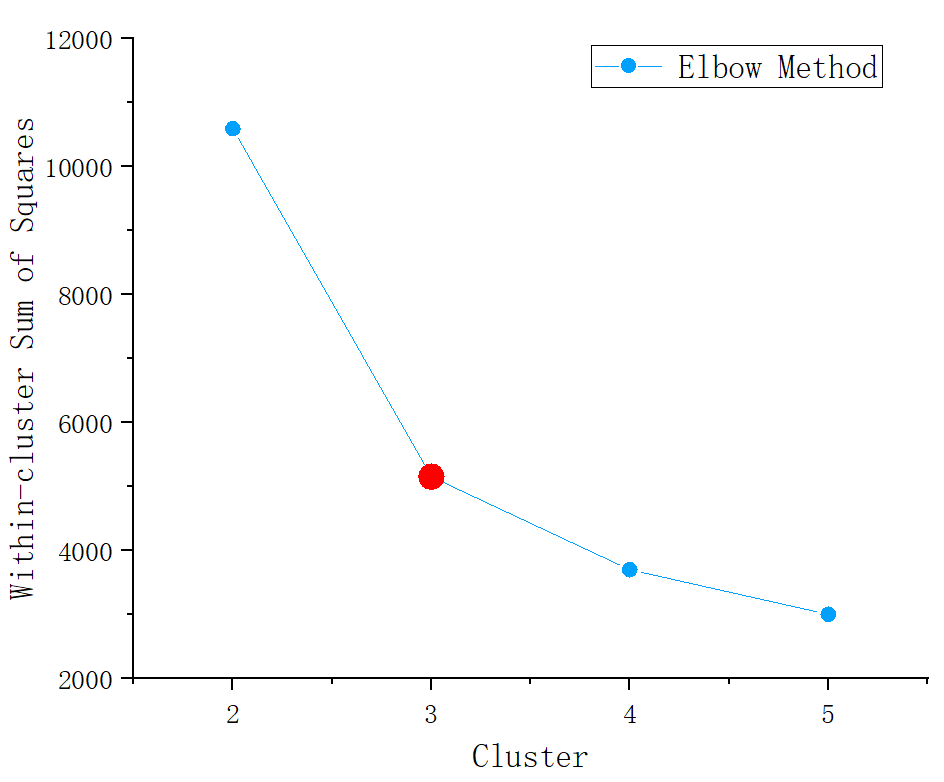

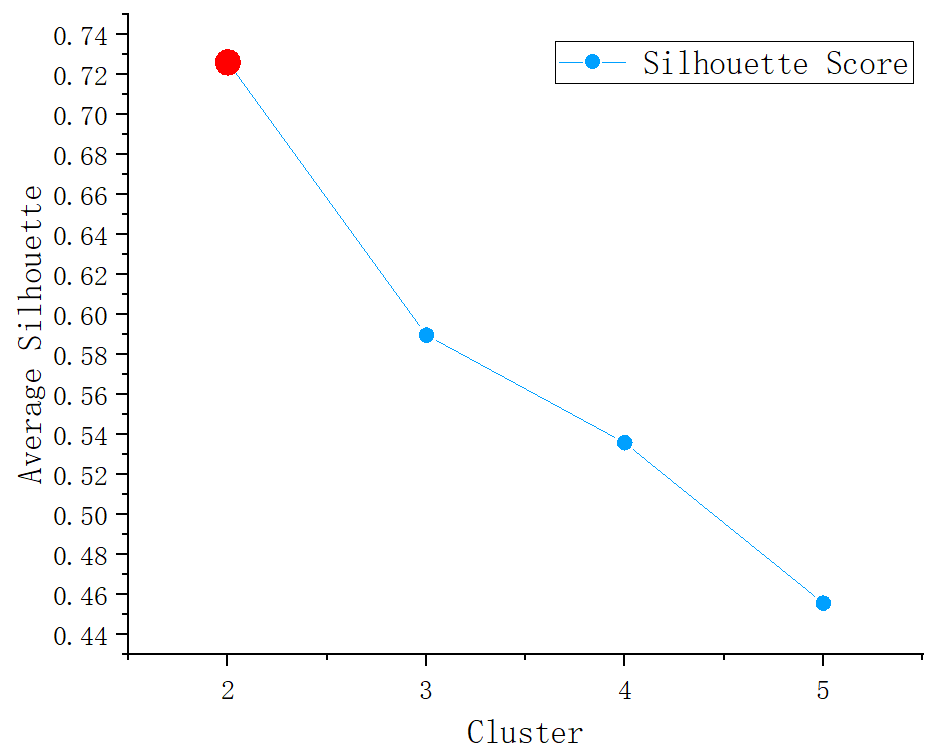


**Fruit skin main color at physiological ripeness**

**Figure S2** The optimal K value for clustering determined by the Elbow Method and Silhouette Coefficient.
