# Supplementary material for: Integrating phenotypic analyses and color parameters: a multidimensional framework for precise color characterization in eggplant fruit
Source: Front Plant Sci. 2025 Dec 10;16:1689896. doi: 10.3389/fpls.2025.1689896 (PMC12728077; doi:10.3389/fpls.2025.1689896)
Supplement: Supplementary file 2 [file Table1.docx]

| **Characteristics** | **The characteristic state and corresponding notes** | | | | | | | | |
| --- | --- | --- | --- | --- | --- | --- | --- | --- | --- |
|  | **1** | **2** | **3** | **4** | **5** | **6** | **7** | **8** | **9** |
| Anthocyanin coloration of hypocotyl (ACH) | Very weak | Very weak to weak | Weak | Weak to medium | Medium | Medium to strong | Strong | Strong to very strong | Very strong |
| Anthocyanin coloration of stem (ACS) | Very weak | Very weak to weak | Weak | Weak to medium | Medium | Medium to strong | Strong | Strong to very strong | Very strong |
| Intensity of leaf blade green color (ILG) | Very light | Very light to light | Light | Light to medium | Medium | Medium to dark | Dark | Dark to very dark | Very dark |
| Anthocyanin coloration of leaf vein (ACL) | Very weak | Very weak to weak | Weak | Weak to medium | Medium | Medium to strong | Strong | Strong to very strong | Very strong |
| Flowers color (FC) | White | Light violet | Violet |  |  |  |  |  |  |
| Fruit pulp color (FPC) | White | Light green |  |  |  |  |  |  |  |
| Fruit skin main color at harvest maturity (FSCH) | White | Green | Purple |  |  |  |  |  |  |
| Intensity of fruit skin main color (IFC) | Very light | Very light to light | Light | Light to medium | Medium | Medium to dark | Dark | Dark to very dark | Very dark |
| Fruit glossiness (FG) | Very weak | Very weak to weak | Weak | Weak to medium | Medium | Medium to strong | Strong | Strong to very strong | Very strong |
| Fruit skin color at physiological ripeness (FSCR) | Yellow | Ochre | Brown |  |  |  |  |  |  |

**Table S1** The value assignment criteria of characteristics related to color and anthocyanin coloration in the eggplant DUS test

Table S2 Sequence of 24 SSR primers used for eggplant varieties identification

| **No.** | **Code** | **linkage group** | **Forward primer (5ʹ-3ʹ)** | **Reverse primer (5ʹ-3ʹ)** | **Recommended fluorescent labeling** | **annealing temperature / ℃** |
| --- | --- | --- | --- | --- | --- | --- |
| 1 | emh02A04 | 1 | ATTGATTTCTAAGCGCACTCGCAC | GTTTAGGGATTGTTCAATTCTGGGTCTG | 5′FAM | 55 |
| 2 | emg11B20 | 1 | ATCATTGCCGTATCAGGTTCACTC | GTTTGGGAAAGTTGAGAATTTCTTGGGG | 5′HEX | 55 |
| 3 | emg11D05 | 2 | ACGTGTGAACTTAAGCAGAATGCTC | GTTTGCAAAAAGTTTCTGTCATGCTCCA | 5′ROX | 55 |
| 4 | emb01J19 | 2 | GACAGGGATAGGGGTACGGATAGG | ATCCATGTGATGCCTCGATTTTCT | 5’FAM | 56 |
| 5 | eme07D02 | 3 | GTGACTGATGAGGTGTTCATCGCT | GTGCCAAACAAGATGCACTTATGG | 5′ROX | 56 |
| 6 | emx30903 | 3 | TCCAAGGAACCTGACCTTAAAT | GGGTACATCACGCAAAGTGATA | 5’FAM | 58 |
| 7 | emh01O20 | 4 | ACTGCAATATTTGGGTTGCAGAGA | GTTTGAGGTCATTTGGTGGTTCAGGT | 5’TAMRA | 55 |
| 8 | emx40203 | 4 | AAAATGTGCTAATACATCTCAAGACA | AACACACCACATGATCCTCATT | 5’FAM | 56 |
| 9 | emx50804 | 5 | TTCCTCAACTAAAATATTAATCGCAT | TGTGTCTACTTCAATTTTGAATCTCTC | 5’HEX | 55 |
| 10 | emx50304 | 5 | GCTGAAGATTGTGGAGGGAATA | TGCAAGCTATTGCATAGGACAG | 5’TAMRA | 56 |
| 11 | emx60604 | 6 | TGTCCTACCATTACACGAATGT | AGAAACAACATAATAGCGTGGAA | 5’TAMRA | 60 |
| 12 | emx61102 | 6 | AGCACCTGTGAGTTCTCTAGGC | GGAATTTGTGGTGTCAAATGAA | 5'ROX | 64 |
| 13 | emf01E17 | 7 | CGAAGGACATCGAGAAAGGGAGTA | GCTATCTTTCCTGCAATCTTTTGCT | 5’TAMRA | 56 |
| 14 | emx70203 | 7 | TCACCTCTGTTCAGTTTGTGCT | TGACTCAAAATGGTGTTCTTCG | 5’TAMRA | 56 |
| 15 | emh02E08 | 8 | AGGCGTTCAGCAGAGAAGAAATTA | GTTTGCTTCCTTAAGTGGCATCTGAAA | 5’FAM | 55 |
| 16 | emx30204 | 8 | ACTCTTATTGTCTAAAATGGTGCAT | TCCTCTCATCTAACAAAGGGTG | 5’TAMRA | 58 |
| 17 | emx81003 | 9 | GACAATAGATGCGATATGAATACGA | CTGTAAATTAAATGGCGCAACA | 5’ROX | 58 |
| 18 | emx91003 | 9 | ATCCCATGAGAAGCAGGTACAG | ATGTGCTTCATTCCCTTTTCTG | 5’HEX | 58 |
| 19 | emxA0405 | 10 | ACAGACAGACAGAGAGGGAAGG | ACAGTGCCAGTGGAGTCTAGGT | 5’FAM | 55 |
| 20 | emxA0703 | 10 | TGATTCTTGAAATCCTATTAGGGC | TGAGTTGGACGGGTTTAATATG | 5’TAMRA | 60 |
| 21 | emxB1002 | 11 | TGACACATTCATTCATTCATGG | CAAGCACAAGCTGTAAGACCTAA | 5’HEX | 58 |
| 22 | emh11j22 | 11 | CTGTGACACATTCATTCATTCATGG | TTGCGTAATGTGCTGATTGACAAG | 5’FAM | 55 |
| 23 | emxC0805 | 12 | GGATGCCTTAAATTCCCACATA | GCAAATCTGGTTCATCAAAGT | 5’ROX | 60 |
| 24 | emxC1103 | 12 | ATTCCACATGATTTTCCAGAGG | ATGCATGAAATTCAGAGCACAA | 5’FAM | 58 |
